# Effect of Nicotinamide Riboside Against the Exhaustion of CD8^+^ T Cells via Alleviating Mitochondrial Dysfunction

**DOI:** 10.3390/nu16213577

**Published:** 2024-10-22

**Authors:** Ying Xiao, Nengzhi Pang, Sixi Ma, Mengqi Gao, Lili Yang

**Affiliations:** Guangdong Provincial Key Laboratory of Food, Nutrition, and Health, Department of Nutrition, School of Public Health, Sun Yat-sen University, Guangzhou 510080, China

**Keywords:** nicotinamide ribose, CD8^+^ T cells exhaustion, mitochondrial

## Abstract

**Background**: Targeting mitochondria and protecting the mitochondrial function of CD8^+^ T cells are crucial for enhancing the clinical efficacy of cancer immunotherapy. **Objectives**: In this study, our objective was to investigate the potential of nicotinamide riboside (NR) in preserving the mitochondrial function of CD8^+^ T cells and mitigating their exhaustion. **Methods**: We established two in vitro models to induce CD8^+^ T cell exhaustion either by tumor cell-conditioned medium (TCM) or by continuous stimulation with OVA_(257–264)_ peptide. CD8^+^ T cells were treated in the absence/presence of NR. **Results**: Our findings demonstrated that NR supplementation effectively inhibited CD8^+^ T cell exhaustion and preserved mitochondrial function in both models. Moreover, apoptosis of CD8^+^ T cells was reduced after NR treatment. Western blot data indicated that NR treatment upregulated Silent information regulator 1 (SirT1) expression. Further inhibition of Sirt1 activity using EX527 uncovered that the inhibitory effect of NR on CD8^+^ T cell exhaustion and its protective effect on mitochondria were attenuated. **Conclusions**: In conclusion, our results indicate that NR supplementation attenuates CD8^+^ T cell exhaustion, and its underlying mechanism is associated with increased mitochondrial function regulated by the SirT1 pathway. Our research provides evidence that NR may assist in enhancing the clinical efficacy of immunotherapy.

## 1. Introduction

Cancer is one of the most important public health problems that threatens the health of human beings [1]. The emergence of immunotherapeutic strategies offers new opportunities for the long-term prevention of the development and recurrence of cancer [2]. Immune checkpoint blockade (ICB) and chimeric antigen receptor T-cell (CAR-T) therapy are two successful strategies for treating patients with many types of cancer in the clinic [2]. However, both of them have certain limitations. Inhibition of CD8^+^ T cell exhaustion is critical for the success of CAR-T cell therapy in treating solid cancers and preventing recurrence, as well as for the effectiveness of ICB applications [3,4,5]. Therefore, it is imperative to develop new strategies to prevent CD8^+^ T cell exhaustion and exploit its therapeutic potential.

A variety of factors in the tumor microenvironment (TME), including persistent antigenic stimulation, cytokine, hypoxia, nutrient deprivation, and exposure to immunosuppressive molecules, affect CD8^+^ T cell differentiation and lead to CD8^+^ T cell exhaustion [6]. CD8^+^ T cells exhibit significant metabolic flexibility [7]. Mitochondria, which are central hubs of metabolism and signaling, play a crucial role in determining CD8^+^ T cell differentiation [8,9,10]. It has been reported that CD8^+^ T cell exhaustion in the TME exhibits mitochondrial dysfunction, including mitochondrial mass and depolarized mitochondrial membrane potentials, and mitochondrial dysfunction could reinforce CD8^+^ T cell exhaustion through various pathways [9,11,12]. Targeting mitochondria in CD8^+^ T cells could mitigate the exhaustion phenotype, leading to enhanced anti-tumor immunity [11,12].

Nicotinamide adenine dinucleotide (NAD) is widely involved in the biochemical reactions of cells, including energy metabolic pathways and substrate for signaling enzymes such as sirtuins, Poly (adenosine diphosphate-ribose) polymerases (PARPs), and cyclic ADP-ribose (cADPR) [13]. Research suggests that decreased NAD is correlated with CD8^+^T cell exhaustion [14]. NR, a precursor to NAD, can increase NAD levels in brown fat, skeletal muscle, and liver [15,16,17]. Silent information regulator 1 (SirT1) is a NAD-dependent deacetylase; the level of NAD affects the deacetylation activity of SirT1. SirT1 can regulate cellular metabolism and mitochondrial biosynthesis by enhancing peroxisome proliferator-activated receptor gamma-coactivator 1-alpha (PGC-1alpha) activity through deacetylation [18]. A recent study demonstrated that in a septicemia mouse model, NR intervention inhibited CD4^+^ T cell exhaustion and elevated SirT1 expression levels [19]. It remains unclear whether the impact of NR on CD8^+^ T cell exhaustion is linked to the activation of SirT1.

Therefore, we aimed to investigate whether the impact of NR on CD8^+^ T cell exhaustion and its underlying mechanisms are associated with mitochondrial function regulated by the SirT1 pathway. The results suggest that NR supplementation may be a promising therapeutic strategy to prevent CD8^+^ T cell exhaustion.

## 2. Materials and Methods

### 2.1. Mice

TCR-transgenic OT-I mice at 4–6 weeks of age were purchased from Changzhou Cavens Laboratory Animal Company (Jiangsu, Changzhou, China). All mice were housed in a certified barrier facility of Sun Yat-sen University and were kept in a 12-h dark/light cycle. All animal experiments in this study were approved by the Animal Care and Protection Committee of Sun Yat-sen University (No. 2023001184).

### 2.2. Tumor Cell Culture

Murine hepatoma cell line Hepa1-6 was obtained from the American Type Culture Collection (ATCC). Hepa1-6 cells were transduced to express luciferase-OVA. Cells were cultured in Dulbecco’s Modified Eagle Medium (DMEM) (Gibco^TM^, Thermo Fisher Scientific Inc., Waltham, MA, USA) supplemented with 10% fetal bovine serum (FBS) (Gibco^TM^, Thermo Fisher Scientific Inc., Waltham, MA, USA) and 1% penicillin-streptomycin (Gibco^TM^, Thermo Fisher Scientific Inc., Waltham, MA, USA). All cells were grown at 37 °C in a 5% CO_2_ incubator.

### 2.3. Preparation of TCM

Hepa1-6-OVA-Luc cells were cultured in RMPI 1640 (Gibco^TM^, Thermo Fisher Scientific Inc., Waltham, MA, USA) supplemented with 10% FBS (Gibco^TM^, Thermo Fisher Scientific Inc., Waltham, MA, USA) and 1% penicillin-streptomycin (Gibco^TM^, Thermo Fisher Scientific Inc., Waltham, MA, USA) for 3 days. The cell culture supernatant was harvested and mixed with T-cell culture medium at a ratio of 1:1.

### 2.4. CD8^+^ T Cells Sorting

Fresh spleens from OT-I mice were mechanically crushed and filtered through a 70-μm strainer (Thermo Fisher Scientific Inc., Waltham, MA, USA). Splenocytes were resuspended in MACS buffer, PBS (Boster, Wuhan, China) supplemented with 0.5%BSA (Beyotime Biotech Inc, Shanghai, China) and 2 mM EDTA (Beyotime Biotech Inc, Shanghai, China). Subsequently, CD8^+^ T cells (ovalbumin-specific T cell receptor (TCR) transgenic T cells) were purified by MACS using the CD8a^+^ T Cell Isolation Kit (Miltenyi Biotec Inc, Cologne, Germany).

### 2.5. Continuous Stimulation of Antigens In Vitro

CD8^+^ T cells were purified from the spleens of OT-I mice. In each well of a12-well plate, 5–10 × 10^5^ of the purified CD8^+^ T cells/mL were cultured in complete media (RPMI 1640, 10% FBS, 1% penicillin-streptomycin, 100 IU/mL IL-2 (Peprotech, Cranbury, NJ, USA), 5 μΜ 2-Mercaptoethanol (Gibco^TM^, Thermo Fisher Sentific Inc., Waltham, MA, USA), 2 mM Glutamax (Gibco^TM^, Thermo Fisher Sentific Inc., Waltham, MA, USA)) with or without 0.5 mM NR. 10 μg/mL OVA_(257–264)_ peptide (MCE, Monmouth Junction, NJ, USA) was added on day 1 and day 3. On day 5, Cells were cultured in complete media without peptide stimulation for three days.

### 2.6. Interventions for TCM In Vitro

CD8^+^ T cells were purified from the spleens of OT-I mice. Cells were cultured in the complete media with or without 0.5 mM NR. OVA_(257–264)_ peptide was added on day 1. On day 3, cells were cultured with complete media without any peptide stimulation for four days. On day 7, cells were cultured with TCM for 24 h.

### 2.7. Cell Viability Analysis

Cell viability was detected by the Cell Counting Kit-8 (CCK-8) (Dojindo, Kumamoto Prefecture, Kyushu Island, Japan). Cells were seeded in a 96-well plate at a density of 1 ×  10^4^/well for 24 h. After 24 h, 10 μL CCK-8 solution was added to each well and incubated at 37 °C, 5% CO_2_ for another 3 h. Then, cell viability was measured using a microplate reader (Tecan, Männedorf, Switzerland) and quantified at 450 nm Absorbance^®^.

### 2.8. Flow Cytometry

Cell apoptosis was analyzed using an Annexin V-FITC/PI apoptosis detection kit (Invitrogen, Carlsbad, CA, USA). Cells were washed twice with PBS and resuspended in 1× binding buffer. Annexin V-FITC was added to cell suspension (100 μL) and incubated for 10–15 min at room temperature away from light. Then, a solution of 5 μL PI was added, and cells were incubated at room temperature in the dark for 5 min. Cells were measured on Cytoflex (Beckman Coulter, Brea, CA, USA) using application settings. Data was then analyzed with FlowJo software (Version 10.6.2, Treestar, Ashland, OR, USA).

Cells were collected in 1mLEP tubes for surface marker staining. Cells were washed twice with PBS and resuspended with PBS to prepare a single-cell suspension. Add the antibodies to single cell suspension and incubate at 4 °C for 30 min, followed by live/dead staining with 7-AAD (eBioscience™, Thermo Fisher Sentific Inc., Waltham, MA, USA) for 5 min. Cells were then washed with PBS containing 2%FBS and resuspended in the same buffer for flow cytometry analyses. Data was then analyzed with FlowJo software.

For intracellular cytokines staining, Protein transport inhibitors (eBioscience™, Thermo Fisher Sentific Inc., Waltham, MA, USA) and 10 ng/mL OVA_(257–264)_ peptide were added to the cell culture for 6–8 h. Cells were collected and resuspended to prepare a single-cell suspension. Cells were first stained for Fixable Viability Dye eFluor™ 780 (eBioscience™, Thermo Fisher Sentific Inc., Waltham, MA, USA) for 30 min in the dark at 4 °C. After staining, cells were washed twice with PBS and fixed with IC Fixation Buffer (eBioscience™, Thermo Fisher Sentific Inc., Waltham, MA, USA) at room temperature in the dark for 20–60 min. Next, cells were washed with permeabilization buffer (eBioscience™, Thermo Fisher Sentific Inc., Waltham, MA, USA) and stained for intracellular cytokines for 20–60 min in the dark at room temperature. Cells were then washed with permeabilization buffer and resuspended in the PBS containing 2%FBS for flow cytometry analyses. Data was then analyzed with FlowJo software.

The following antibodies were purchased from eBioscience™: Anti-Mouse CD279 (PD-1) eFluor^®^ 450, Anti-mouse CD366 (Tim-3) APC, Anti-mouse TNF alpha eFluor^®^ 450, Anti-mouse IFN gamma APC.

### 2.9. Measurement of Mitochondrial Membrane Potential

The mitochondrial membrane potential of cells was measured using the JC-1 Mitochondrial Membrane Potential Assay Kit (Beyotime, Shenzhen, China). Cells were washed twice with PBS and then resuspended with cell culture media, and then stained with JC-1 work solution for 20 min in the dark at 37 °C. Next, Cells were washed twice with ice-cold 1× JC-1 staining buffer and resuspended in the PBS containing 2%FBS for flow cytometry analyses.

### 2.10. Measurement of Mitochondrial Reactive Oxygen Species

The mitochondrial reactive oxygen species of cells were measured by MitoSOX™ Red mitochondrial superoxide indicator (Thermo Fisher Sentific Inc., Waltham, MA, USA). Cells were washed twice with PBS and then stained with 5 μmol/L MitoSOX solution for 10 min in the dark at 37 °C. Next, Cells were washed twice with PBS containing 2%FBS and resuspended in the same buffer for flow cytometry analyses.

### 2.11. In Vitro Killing Assay

Hepa1-6-OVA-Luc cells were digested with trypsin, resuspended with T cell culture media as described above, and seeded in 96-well microplates. Each well was seeded with 100 μL of cell suspension containing 10^4^ cells. 100 μL of CD8^+^ T cells were added to the tumor cell culture with different Effector cell-to-target cell ratios (E:T ratio) in 96-well plates and co-culture for 24 h. After 24 h of co-culture, the medium containing CD8^+^ T cells was removed, and the cells were washed twice with PBS. Next, tumor cells were incubated in 100 μL of 1 mmol/L D-Luciferin working solution for 10 min in the dark at 37 °C. Subsequently, Tumor cells were measured using a microplate reader and quantified at 540–600 nm Bioluminescence.

### 2.12. Western Blotting

The cells were lysed in RIPA buffer and supplemented with protease and phosphatase inhibitors. Total proteins were separated via SDS-PAGE and subsequently transferred to polyvinylidene fluoride (PVDF) membranes in equal quantities. Next, the membranes were blocked with 5% fat-free milk for 2 h and then treated overnight at 4 °C with primary antibodies, Sirt1 (Cell Signaling Technology, Danvers, MA, USA), PGC1-α (Cell Signaling Technology, Danvers, MA, USA), Caspase3 (Cell Signaling Technology, Danvers, MA, USA), Ac-lysine (Santa Cruz Biotechnology, Paso Robles, CA, USA), GAPDH (Cell Signaling Technology, Danvers, MA, USA).

The membranes were then washed and incubated at room temperature for 2 h with a secondary antibody, Anti-mouse IgG (Santa Cruz Biotechnology, Paso Robles, CA, USA) and Anti-rabbit IgG (Santa Cruz Biotechnology, Paso Robles, CA, USA). Blots were developed using an ECL kit (Thermo Fisher Sentific Inc., Waltham, MA, USA), and images were captured with the Chemiluminescent Imaging System (Tanon, Shanghai, China).

### 2.13. Statistics

GraphPad Prism 9 software was used for statistical analysis and graphing, and FlowJo 10.6.2 was used for graphing. All data were expressed as mean ± standard error. Data comparisons between two groups were performed by *t*-test, and data comparisons between multiple groups were performed by one-way ANOVA. Comparisons between two or more groups of repeated-measures data were analyzed by two-way ANOVA. *p* < 0.05 indicates a statistically significant difference.

## 3. Results

### 3.1. NR Inhibits CD8^+^ T Cell Exhaustion

**Figure 1:**
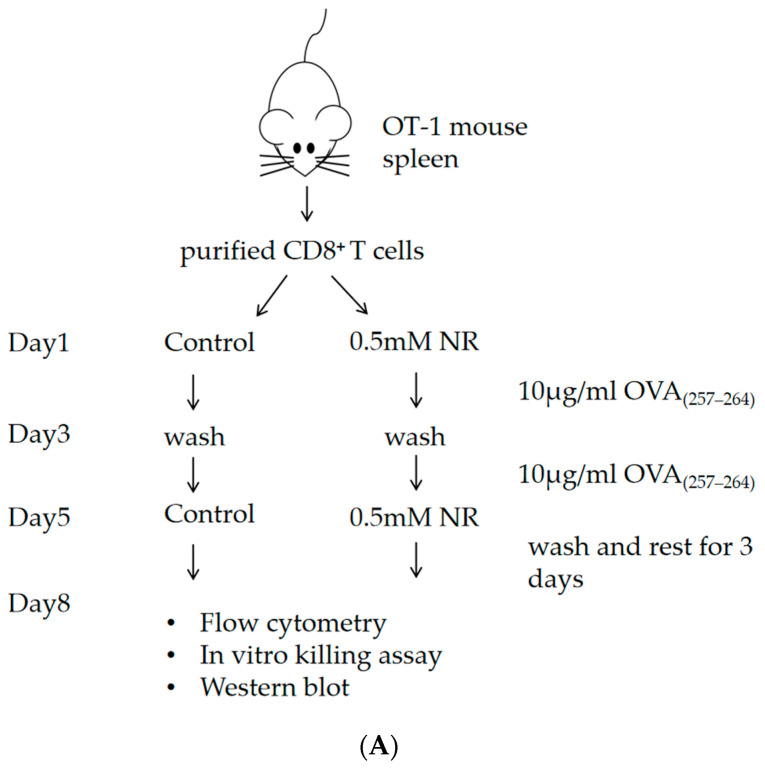

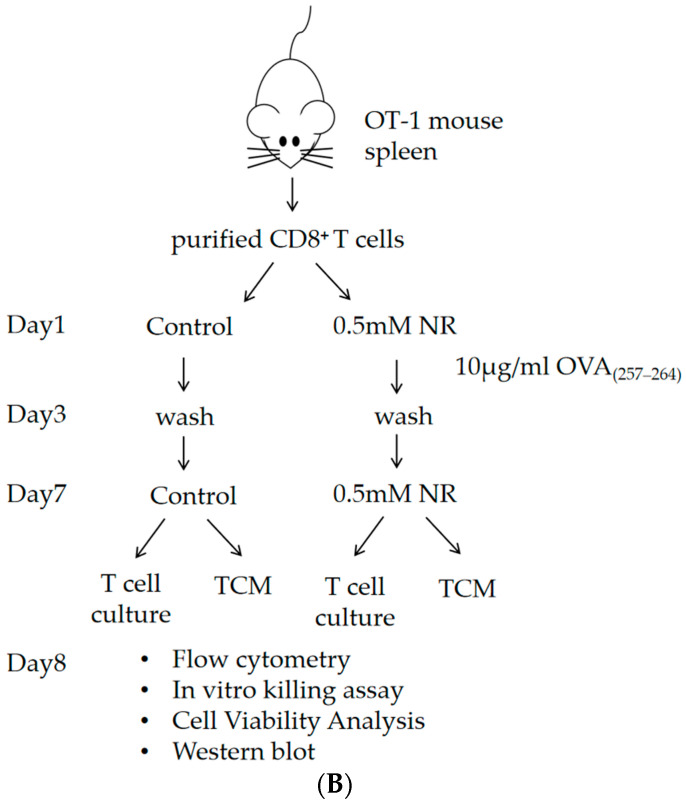
The experimental protocol (**A**) of CD8^+^ T cell exhaustion induced by continuous stimulation with OVA_(257–264)_ peptide, (**B**) of CD8^+^ T cell exhaustion induced by TCM.

**Figure 2:**
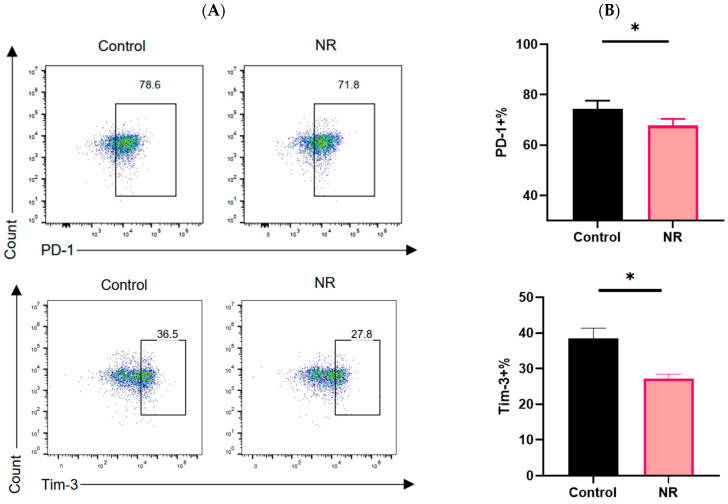

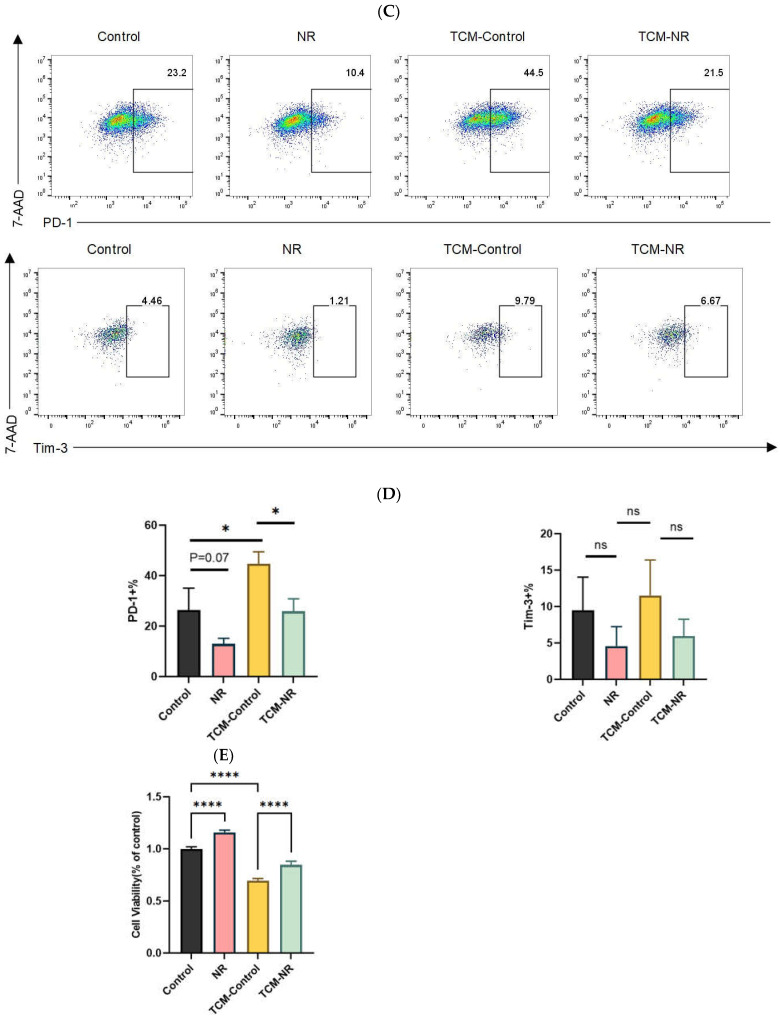
NR inhibits CD8^+^ T cell exhaustion. Under continuous stimulation with OVA_(257–264)_ peptide (**A**) Frequency of expression of inhibitory receptors PD-1 and Tim-3 detected by flow cytometry, (**B**) Statistical analysis of PD-1 and Tim-3 expression frequencies detected by flow cytometry; Under T cell culture medium conditions and TCM conditions (**C**) Frequency of expression of inhibitory receptors PD-1 and Tim-3 detected by flow cytometry, (**D**) Statistical analysis of PD-1 and Tim-3 expression frequencies detected by flow cytometry. (**E**) Graph of statistical results of the CCK-8 Assay for CD8^+^ T Cell Viability. Three independent experiments were performed. The line depicts mean ± SE. * *p* < 0.05, **** *p* < 0.0001.

Studies have shown that CD8^+^ T cell exhaustion shows increased expression of inhibitory receptors [20,21]. Programmed cell death protein 1 (PD-1) is a key depletion marker for CD8^+^ T cells and plays an important role in tumor immunity. T cell immunoglobulin and mucin domain-containing protein 3 (Tim-3) has also been found to be associated with functional depletion of CD8^+^ T cells. Targeting Tim-3 and the PD-1 pathway could reverse T-cell exhaustion [22]. Therefore, we first examined PD-1 and Tim-3 expression in CD8^+^ T cells. Our data showed that under continuous stimulation with OVA_(257–264)_ peptide (Figure 1A), Compared to the control group, the expression of PD-1 in NR-intervened CD8^+^ T cells decreased by 6.53 ± 0.9%, and the expression of TIM-3 decreased by 11.23 ± 2.4% (Figure 2A,B). Under T Cell culture conditions (Figure 1B), Compared to the control group, the expression of PD-1 in NR-intervened CD8^+^ T cells decreased by13.45 ± 5.76%, and the expression of TIM-3 decreased by 4.90 ± 2.38% (Figure 2C,D). In contrast, after 24 h of TCM intervention (Figure 1B), Compared to the control group, the expression of PD-1 in NR-intervened CD8^+^ T cells decreased by18.84 ± 2.77%, and the expression of TIM-3 decreased by 5.57 ± 3.14% (Figure 2C,D). These results indicate that NR supplementation has inhibitory effects on the exhaustion of CD8^+^ T cells. Meanwhile, we observed that CD8^+^ T cells treated with 0.5 mM NR exhibited higher viability compared to untreated CD8^+^ T cells under both T cell culture and TCM culture conditions (Figure 2E). These findings suggest that short-term exposure to 0.5 mM NR does not exert cytotoxic effects on CD8^+^ T cells. Consequently, a concentration of 0.5 mM NR was selected for further experiments.

### 3.2. NR Enhances Cytokine Production and Cytotoxic Function of CD8^+^ T Cells

**Figure 3:**
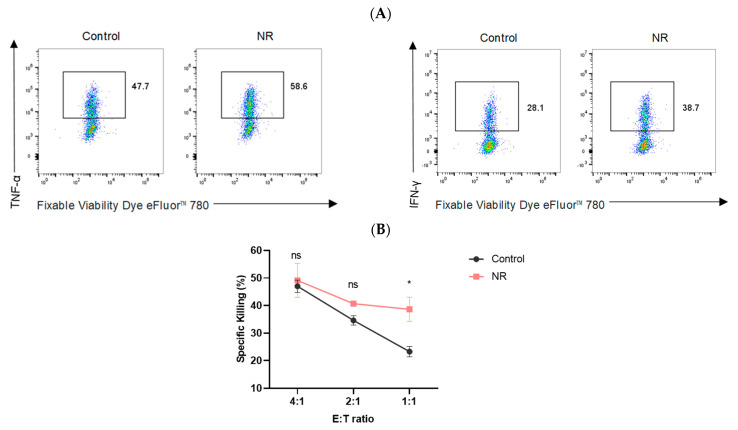

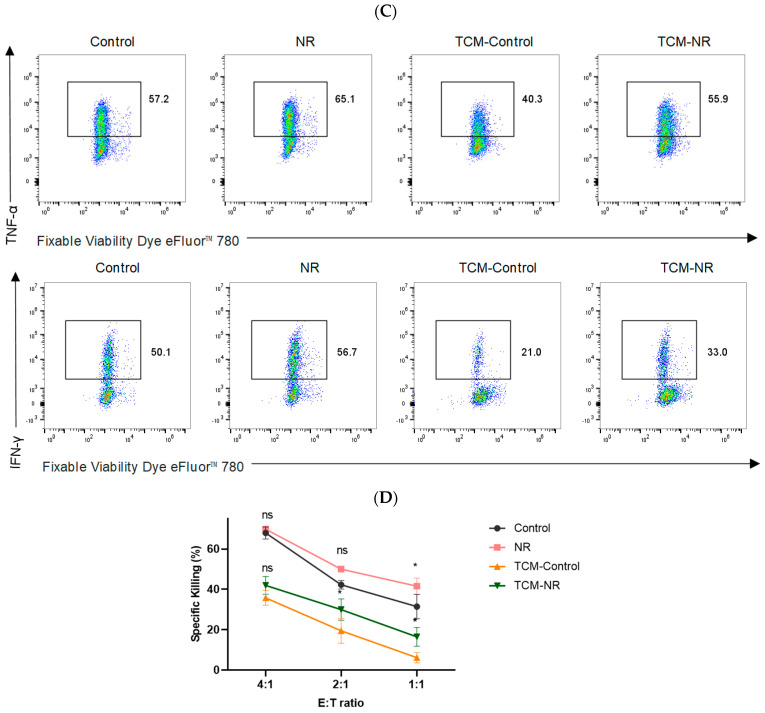
NR enhances cytokine production and Cytotoxic function of CD8^+^ T cells. Under continuous stimulation with OVA_(257–264)_ peptide (**A**) Frequency of expression of TNF-α and IFN-γ detected by flow cytometry, (**B**) CD8^+^ T cells were co-culture with target cells (Hepa1-6-Luc-OVA cells) at different ratios. The percentage of specific killings is depicted.; Under T cell culture medium conditions and TCM conditions (**C**) Frequency of expression of TNF-α and IFN-γ detected by flow cytometry, (**D**) CD8^+^ T cells were co-culture with target cells (Hepa1-6-Luc-OVA cells) at different ratios. The percentage of specific killings is depicted. * *p* < 0.05.

Antigen-activated CD8^+^ T cells produce a variety of cytokines, including IL-2, TNF-α, and IFN-γ, which are essential for antitumor immune responses. During the exhaustion process, CD8^+^ T cells experience a stratified loss of cytokine production capacity, which has an impact on their anti-tumor efficacy [23]. To investigate the effector function of NR on CD8^+^T cells, we first examined the ability to release cytokines after OVA_(257–264)_ peptide restimulation. Our data showed that under continuous stimulation with OVA_(257–264)_ peptide, TNF-α and IFN-γ expression levels are higher in NR-treated CD8^+^ T cells than in non-NR-treated CD8^+^ T cells (Figure 3A). Under T Cell culture conditions, NR-treated CD8^+^ T cells exhibited higher expressions of TNF-α and IFN-γ compared to the non-NR-treated CD8^+^ T cells (Figure 3C). After 24 h of TCM intervention, the expression levels of TNF-α and IFN-γ are higher in NR-treated CD8^+^ T cells than in non-NR-treated CD8^+^ T cells (Figure 3C). In addition, CD8^+^ T cells were co-cultured with Hepa1-6-Luc-OVA cells for 24 h. Following this, an examination of Hepa1-6 Luc-OVA fluorescence intensity was conducted in order to investigate the in vitro killing ability of CD8^+^ T cells. We observed that NR-treated CD8^+^ T cells exhibited a greater ability to kill tumor cells compared to the non-NR-treated CD8^+^ T cells after co-culturing with Hepa1-6-Luc-OVA cells for 24 h under continuous stimulation with OVA_(257–264)_ peptide (*p* < 0.05 at 1:1 ratio) (Figure 3B). Similarly, we observed the same results in the T cell culture conditions (*p* < 0.05 at 1:1 ratio) and in the TCM culture conditions (*p* < 0.05 at 1:1 and 2:1 ratio) (Figure 3D). The above results indicate that NR can effectively enhance the effector function of CD8^+^ T cells.

### 3.3. NR Inhibits CD8^+^ T Cell Apoptosis

**Figure 4:**
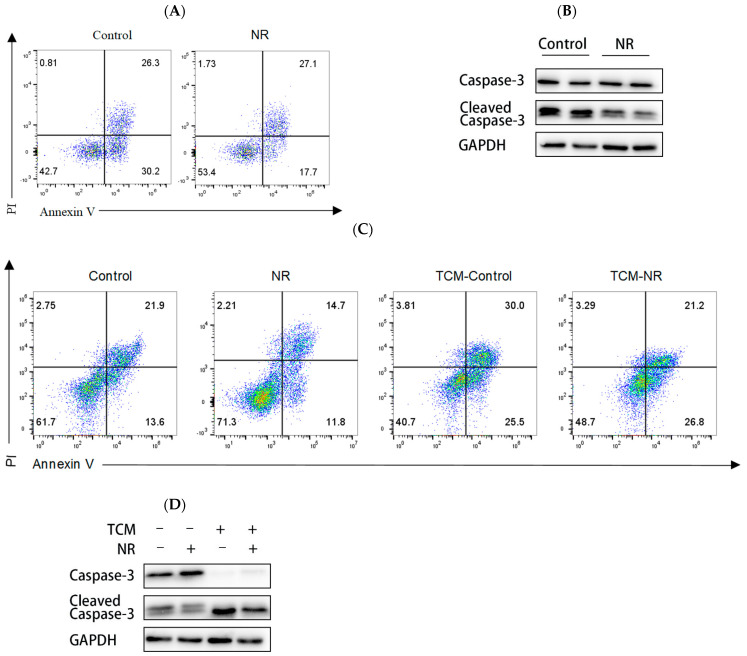
NR increases CD8^+^ T cell viability and inhibits CD8^+^ T cell apoptosis. Under continuous stimulation with OVA_(257–264)_ peptide (**A**) Frequency of expression of cell apoptosis rate detected by flow cytometry, (**B**) Detection of cleaved-caspase3 protein expression in CD8^+^ T cell by Western blot.; Under T cell culture medium conditions and TCM conditions (**C**) Frequency of expression of cell apoptosis rate detected by flow cytometry; (**D**) Detection of cleaved-caspase3 protein expression in CD8^+^ T cell by Western blot.

High expression of multiple inhibitory receptors exacerbates CD8^+^ T cell exhaustion and induces apoptosis [24]. So, we explored the effect of NR on CD8^+^ T cell apoptosis. Our data showed that under continuous stimulation with OVA_(257–264)_ peptide, NR-treated CD8^+^ T cells have lower apoptosis rates than CD8^+^ T cells w/o NR treatment. (Figure 4A). Similarly, we observed the same results in the T cell culture conditions and in the TCM culture conditions (Figure 4C). Cleaved-caspase3 is a key factor in the apoptotic process, responsible for executing the apoptotic program and promoting cell death [25]. We further verified this result by the detection of cleaved-caspase3 protein expression. NR-treated CD8^+^ T cells showed reduced cleaved-caspase3 protein expression under the above intervention conditions (Figure 4B,D). The above results indicate that NR can effectively inhibit CD8^+^ T cell apoptosis.

### 3.4. NR Increases Mitochondrial Membrane Potential and Reduces Mitochondrial Oxidative Stress

**Figure 5:**
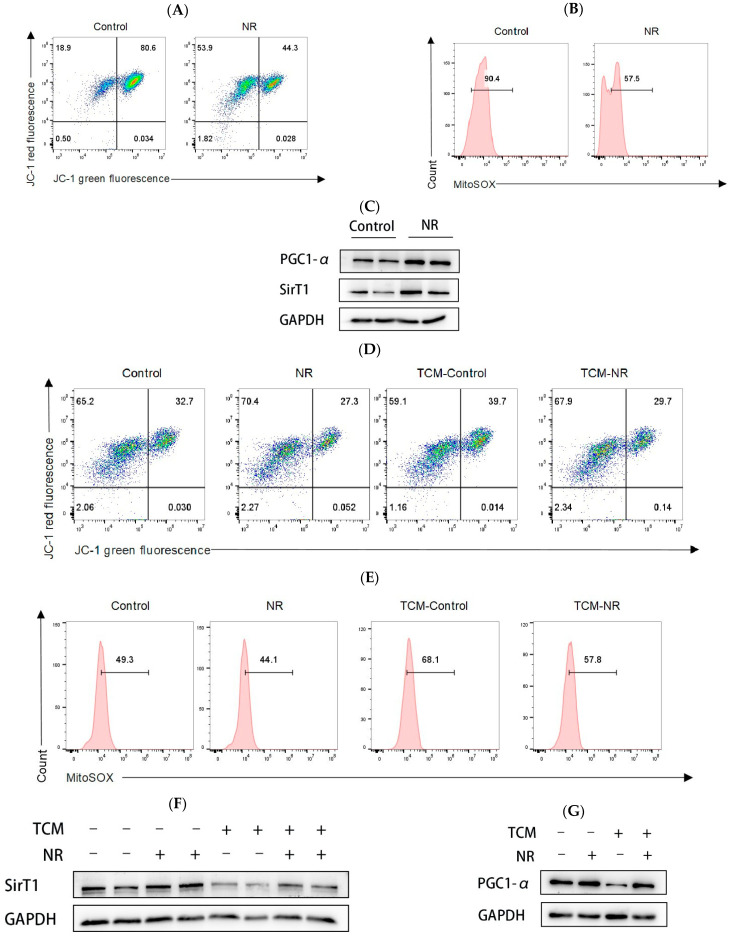
NR increases mitochondrial membrane potential and reduces mitochondrial oxidative stress levels. Under continuous stimulation with OVA_(257–264)_ peptide (**A**) JC-1 levels in CD8^+^ T cells detected by flow cytometry, (**B**) Detection of mtROS expression in CD8^+^ T cell by flow cytometry, (**C**) Detection of Sirt1 and PGC1-α protein expression in CD8^+^ T cell by Western blot; Under T cell culture medium conditions and TCM conditions (**D**) JC-1 levels in CD8^+^ T cell detected by flow cytometry, (**E**) Detection of mtROS expression in CD8^+^ T cell by flow cytometry, (**F**) Detection of Sirt1 protein expression in CD8^+^ T cell by Western blot, (**G**) Detection of PGC1-α protein expression in CD8^+^ T cell by Western blot.

In the tumor microenvironment, exhausted CD8^+^ T cells exhibit inhibition of mitochondrial respiration and aerobic glycolysis, as well as mitochondrial depolarization and excessive mitochondrial ROS production [26]. Mitochondrial dysfunction is believed to be a significant contributor to the induction of CD8^+^ T exhaustion [9,27]. Therefore, the objective of this study was to ascertain whether NR could protect mitochondrial membrane potential and reduce ROS levels. Our data showed that under continuous stimulation with OVA_(257–264)_ peptide, NR-treated CD8^+^ T cells have higher mitochondrial membrane potential (Figure 5A) and less mitochondrial ROS production than CD8^+^ T cells not treated with NR. (Figure 5B). Similarly, we observed the same results in the T cell culture conditions and in the TCM culture conditions (Figure 5D,E). We next examined SirT1 and PGC1-α protein expression levels, which represent an important pathway regulating mitochondrial biosynthesis. We found that NR significantly increased SirT1 and PGC1-α protein expression levels in the continuous stimulation with OVA_(257–264)_ peptide conditions and TCM culture conditions (Figure 5C,F,G). Altogether, these results show that supplementation with NR alleviates mitochondrial dysfunction.

### 3.5. Inhibition of SirT1 Activity Reduces the Protective Effect of NR on CD8^+^ T Cell and Mitochondrial Function

**Figure 6:**
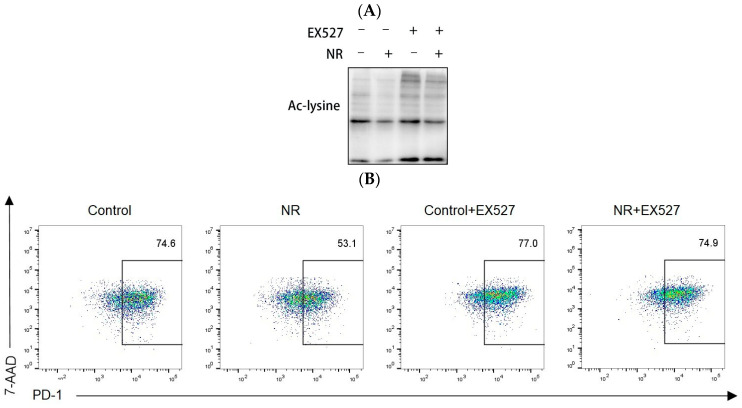

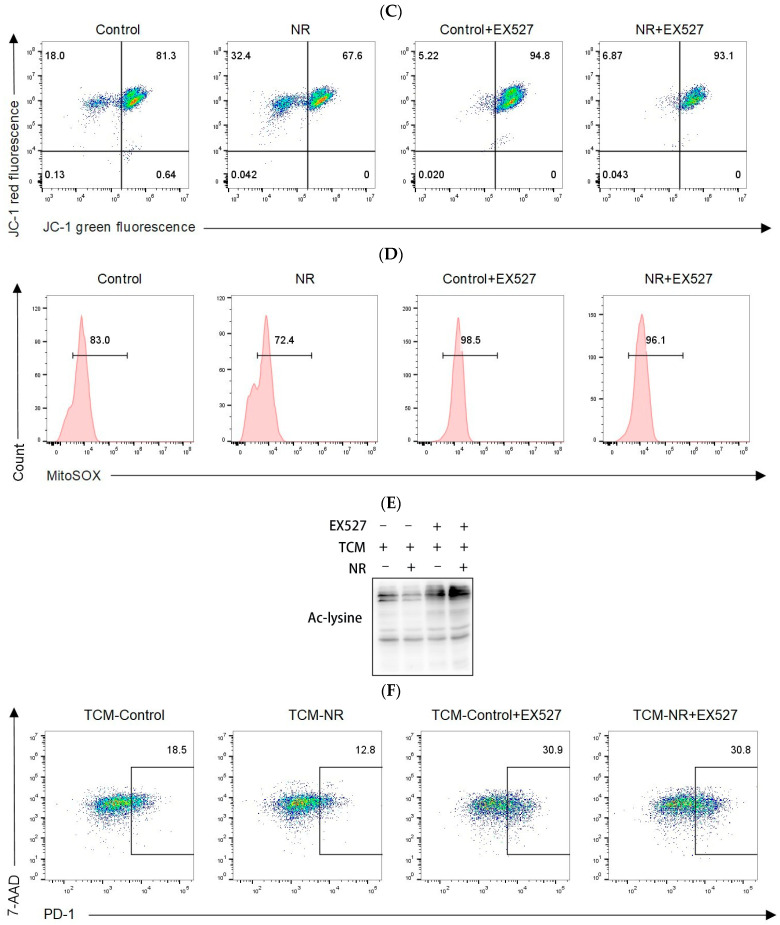

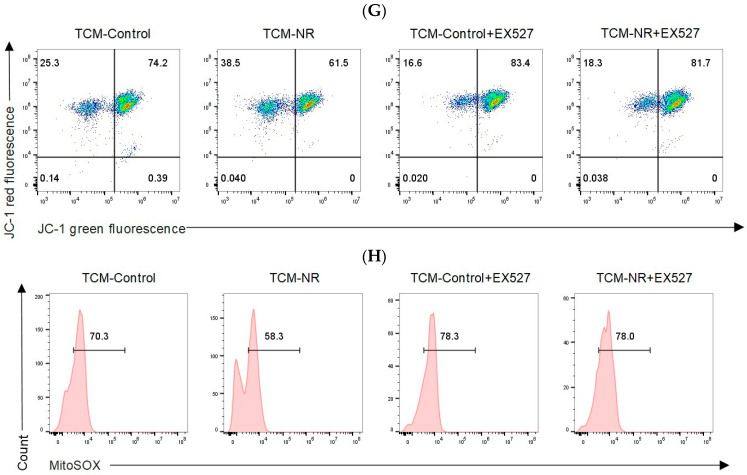
Inhibition of SirT1 activity reduces the protective effect of NR on CD8^+^ T cells and mitochondrial function. Under continuous stimulation with OVA_(257–264)_ peptide (**A**) Detection of acetylated lysine protein expression in CD8^+^ T cell by Western blot, (**B**) PD-1 levels in CD8^+^ T cell detected by flow cytometry, (**C**) JC-1 levels in CD8^+^ T cell detected by flow cytometry, (**D**) Detection of mtROS expression in CD8^+^ T cell by flow cytometry; Under TCM conditions (**E**) Detection of acetylated lysine protein expression in CD8^+^ T cell by Western blot, (**F**) PD-1 levels in CD8^+^ T cell detected by flow cytometry, (**G**) JC-1 levels in CD8^+^ T cell detected by flow cytometry, (**H**) Detection of mtROS expression in CD8^+^ T cell by flow cytometry.

We next examined whether Sirt1 signaling regulates CD8^+^ T cell mitochondrial dysfunction to elucidate the underlying mechanism in the persistent antigen stimulation and TCM intervention. We wanted to investigate the effect of NR on CD8^+^ T cells in the presence of SirT1 inhibition using EX527 to inhibit SirT1 activity. To verify the inhibition of SirT1 activity by EX527, we first examined the protein levels of acetylated lysine (Ac-lysine) in CD8^+^ T cells. Our data show that the control+ EX527 groups and NR+ EX527 groups have increased Ac-lysine protein levels (Figure 6A,E). The above results suggest that EX527 can inhibit SirT1 activity in CD8^+^ T cells. We next assessed whether PD-1 expression could be reduced in the presence of sirt1 activity inhibition. We found that NR significantly reduced PD-1 expression, and inhibiting SirT1 activity reduced the inhibitory effect of NR on PD-1 expression (Figure 6B,F). We simultaneously examined mitochondrial membrane potential and mtROS levels. We found that NR significantly increased mitochondrial membrane potential and reduced ROS levels, and inhibiting SirT1 activity reduced the protective effect of NR on mitochondrial membrane potential and inhibitory effects on mtROS production (Figure 6C,D,G,H). Collectively, these findings reveal that supplementation with NR alleviates mitochondrial dysfunction and CD8^+^ T cell exhaustion by regulating SirT1 activity.

## 4. Discussion

CD8^+^ T cell exhaustion is a significant barrier in cancer immunotherapy [28]. To reactivate exhausted CD8^+^ T cells, various strategies have been developed, such as targeting mitochondria to reduce mitochondrial oxidative stress and reshape mitochondrial metabolism [29]. In this study, we used TCM intervention to mimic the in vitro tumor microenvironment and sustained stimulation with OVA_(257–264)_ to create a CD8^+^T cell exhaustion model and supplemented CD8+T cells with NR to investigate its role in CD8+T cell exhaustion. Our study uncovered that NR inhibited CD8^+^ T cell exhaustion and increased the cytotoxicity of CD8^+^ T cells to liver cancer cells. The inhibition of exhaustion of CD8+T cells is partly through increasing mitochondrial function via Sirt1.

CD8^+^ T cells play a pivotal role in tumor immunity. Upon antigen stimulation, CD8^+^ T cells can rapidly differentiate into effector cells and produce a multitude of effector cytokines (e.g., IL-2, TNF-α, and IFN-γ) and granzyme B to effectively eliminate tumor cells. Conversely, various factors within the tumor microenvironment, such as continuous antigenic stimulation, hypoxia, and lactic acid accumulation, lead to the exhaustion of CD8^+^ T cells [30]. Existing evidence indicates that elevated CD38 (NAD-consuming enzymes) expression is a feature of an impaired CD8^+^ T cell immune response in a metastatic pleural effusion disease model [31]. In a melanoma tumor model, CD38 was similarly found to be highly expressed in exhausted CD8^+^ T cells and showed a correlation with ICB resistance. The blockade of CD38 has been demonstrated to restore cellular NAD levels, improve mitochondrial function, increase proliferation, enhance effector function, and restore ICB sensitivity [32]. NR acts as a precursor to NAD, which can increase NAD levels and alleviate a number of diseases [33]. This suggests that NR may have the potential to inhibit CD8^+^ T cell exhaustion. This is supported by our results showing that supplementation with NR reduced the expression levels of PD-1 and Tim-3 in CD8^+^ T cells. We also observed that NR could increase the secretion levels of TNF-α and IFN-γ, suggesting that CD8^+^ T cells may have a stronger ability to kill tumor cells. Therefore, we directly co-cultured CD8^+^ T cells with Hepa1-6-OVA-Luc for 24 h to determine the tumor cell-killing ability of CD8^+^ T cells. Our results showed a greater ability to kill tumor cells in the presence of NR. Our study provides strong evidence for the use of NR as a nutrient to support immunotherapy.

The presence of hypoxia, nutrient deprivation, and inhibitory receptor expression in the tumor microenvironment leads to mitochondrial dysfunction and metabolic abnormalities, which in turn promote CD8^+^ T cell exhaustion [9,11,12,34,35]. Emerging evidence indicates modulating mitochondria can increase the efficacy of immunotherapy [36]. Several studies reported that NR enhances mitochondrial function [17,37,38]. Consistent with these findings, our results imply that NR increased the mitochondrial membrane potential and decreased mtROS production in CD8^+^ T cells.

Upon encountering antigenic stimulation, CD8^+^ T cells utilize oxidative phosphorylation (OXPHOS) and glycolytic metabolic forms to generate energy and exert their cytotoxic functions [39]. The level of PGC-1α, a key regulator of mitochondrial biogenesis and cellular metabolism, is contingent upon the energy demands. During the activation of CD8^+^ T cells, the expression of PGC-1α is upregulated, which enhances mitochondrial function and supports the cell’s increased metabolic needs. Mitochondrial metabolic dysfunction in tumor-infiltrating CD8^+^ T cells has been linked to the downregulation or suppression of PGC-1α [26]. Notably, a study has shown that enforced PGC-1α expression in CD8 T cells promotes their persistence and memory formation and enhances antitumor immunity [11]. This suggests that strategies to enhance PGC-1α activity could be beneficial for improving T cell-based immunotherapies. However, it has not yet been reported whether NR supplementation can increase the expression of PGC-1α in CD8^+^ T cells. Our results show that PGC-1α protein expression was increased under both TCM intervention and continuous stimulation with OVA_(257–264)_ peptide.

Deacetylation of PGC-1α by SirT1, a nuclear protein, is widely associated with metabolic control and mitochondrial biosynthesis [18,40]. The level of NAD affects the deacetylation activity of SirT1, and a decrease in its level may lead to a decrease in SirT1 activity, which may, in turn, affect the functional and metabolic state of the cell. Our findings indicated that NR increases the protein expression of SirT1. Furthermore, we sought to ascertain the impact of NR on CD8^+^ T cells in the context of SirT1 activity inhibition. Our findings that the inhibitory effect of NR on CD8^+^ T cell exhaustion and the protective effect of mitochondria were reduced in the presence of SirT1 activity inhibition confirmed an important role of SirT1 on the protective effects of NR on CD8^+^ T cells.

Hormesis is a biphasic dose-response phenomenon that describes the positive adaptive response exhibited by organisms when subjected to low-dose stimuli, while higher doses elicit inhibitory effects. Nutritional supplements, such as polyphenols and trace elements, which are also referred to as hormetic nutrients, can stimulate adaptive responses in organisms at low doses, thereby enhancing their stress resistance and promoting health [41,42]. Given that Nicotinamide Riboside (NR) can activate the Sirt1 pathway and improve mitochondrial function at low/medium doses (0.5 mM), it is also considered a stimulating nutrient.

## 5. Conclusions

In summary, NR administration studies provide sufficient in vitro evidence for effective CD8^+^ T cell anti-exhaustion interventions with NR. Our findings emphasize that the CD8^+^ T cells, after NR intervention, have a greater killing capacity in vitro. However, this study has certain limitations. The conclusions of this study were only validated with in vitro cellular experiments and could not be extrapolated to in vivo studies or clinical population studies. Whether NR can inhibit tumor progression by affecting the function of CD8^+^ T cells, need to be further verified. Our research group has previously demonstrated that NR can effectively inhibit the progression and metastasis of Hepatocellular Carcinoma (HCC) in animal models. Additionally, in vitro studies have confirmed NR’s ability to suppress the invasiveness and migratory behavior of HepG2 cells [15]. Moving forward, it is imperative to determine whether the in vitro effects of NR on CD8^+^ T cells are mirrored in vivo. To this end, establishing an animal tumor model will be crucial for assessing immune cell infiltration and CD8^+^ T cell exhaustion markers within tumor tissues after NR intervention. Upon successful validation of the animal model, subsequent clinical population studies can be initiated. These studies will be pivotal in evaluating the combined efficacy of immunotherapy and NR, focusing on key outcomes such as overall survival, objective remission rates, and progression-free survival in patients. The current investigation has delineated the pivotal role of SirT1 signaling in the modulation of mitochondrial functionality; however, the underlying regulatory mechanisms have yet to be fully elucidated. It is, therefore, imperative that further mechanistic dissection be carried out in order to elucidate the intricate pathways through which SirT1 exerts its effects on mitochondrial dynamics. Concurrently, it is imperative to expand the scope of the inquiry to encompass additional molecular determinants and signaling pathways that may contribute to T cell exhaustion beyond the scope of SirT1.

## Data Availability

The original contributions presented in the study are included in the article, further inquiries can be directed to the corresponding author. The data are not publicly available due to privacy restrictions.
